# Impact of the herbal medicine, *Ephedra sinica* stapf*,* on gut microbiota and body weight in a diet-induced obesity model

**DOI:** 10.3389/fphar.2022.1042833

**Published:** 2022-11-15

**Authors:** Eun-Ji Song, Na Rae Shin, Songhee Jeon, Young-Do Nam, Hojun Kim

**Affiliations:** ^1^ Research Group of Personalized Diet, Korea Food Research Institute, Wanju-gun, Jeollabuk-do, South Korea; ^2^ Department of Rehabilitation Medicine of Korean Medicine, Dongguk University, Goyang-si, Gyeonggi-do, South Korea; ^3^ Department of Biomedical Sciences, Center for Global Future Biomedical Scientists at Chonnam National University, Gwangju, South Korea

**Keywords:** obesity, anti-obesity drug, gut microbiota, bupropion, Ephedra sinica

## Abstract

Obesity is a chronic metabolic disease caused by excessive body fat and has become a global public health problem. Evidence suggests that obesity and obesity-induced metabolic disorders are closely related to gut microbiota. Bupropion (BP), an antidepressant medicine, and *Ephedra sinica* Stapf [Ephedraceae; Ephedrae Herba], a herbal medicine, are sympathetic stimulants and have weight loss effects. However, to our best knowledge, no studies have simultaneously assessed the effects of drugs and herbal medicines on obesity and gut microbiota. This study aimed to determine the effects of BP and ES on weight loss and re-modulation of host gut microbiota. To test this hypothesis, we fed C57BL/6J mice with a high-fat diet supplemented with bupropion (BP; 30 mg/kg/day) and *Ephedra sinica* Stapf extract (ES; 150 mg/kg/day) *via* oral gavage for eight weeks. Further, we evaluated the effects of BP and ES on body weight and fat accumulation. In addition, we evaluated the effects of BP and ES on gut microbiota using 16S rRNA amplicon sequencing. Our results showed that weight loss was confirmed in both BP and ES; however, it was more pronounced in ES. ES changed the overall composition of the gut microbiota by restoring the relative abundance of *Oscillospiraceae*, *Lachnospiraceae*, and the *Firmicutes*/*Bacteroidetes* ratio, an indicator of gut microbiota dysbiosis. Nine amplicon sequence variants (ASVs) of the gut microbiome were significantly recovered by BP and ES treatment, of which eight ASVs correlated with body weight and fat accumulation. Additionally, three ASVs were significantly recovered by ES treatment alone. In conclusion, the anti-obesity effects of BP and ES, especially fat accumulation, are related to the regulation of gut microbiota. Moreover, ES had a greater influence on the gut microbiota than BP.

## 1 Introduction

Overweight and obesity are global public health problems because obesity is related to chronic, systemic inflammation, leading to insulin resistance and ultimately type 2 diabetes (Rohm et al., 2022). Increased energy intake and reduced expenditure are the primary driving forces of obesity, and changes in exercise or dietary composition are recommended for weight loss (Romieu et al., 2017). Moreover, depending on the severity, prescription drugs or surgery is also recommended for the treatment of obesity (Nudel and Sanchez, 2019; Müller et al., 2022).

Herbal medicines are considered a common alternative therapy worldwide based on a thousand-year history and phenotype-based clinical trials (Li and Weng, 2017). Numerous studies have been conducted on the effectiveness and safety of herbal medicines for obesity and metabolic syndromes (Payab et al., 2020). Green tea extract reduces weight, waist circumference, total cholesterol, and low-density lipoprotein (LDL) plasma levels in women with central obesity (Chen et al., 2016). Extract of *Hibiscus sabdariffa* L. [Malvaceae] reduced obesity, abdominal fat, and serum free fatty acid (FFA) as well as improved liver steatosis in obese individuals (Chang et al., 2014). Cinnamon consumption has resulted in substantial improvements in all components of the metabolic syndrome in Asian Indians (Jain et al., 2017). *Ephedra sinica* Stapf [Ephedraceae; Ephedrae Herba] (ES), a traditional Chinese botanical drug, has sympathomimetic effects and has been used to treat colds, arthralgia, edema, and asthma (Rotblatt and Ziment, 2002). Moreover, ES is effective in reducing body weight, body mass, and body fat percentage in women with obesity (Kim et al., 2008; Kim et al., 2014). The *Ephedra* and caffeine mixture improves metabolic parameters such as heart rate, serum cholesterol, triglycerides, glucose, and fasting insulin in overweight and obese premenopausal women (Hackman et al., 2006).

Herbal medicines exert anti-obesity effects *via* various mechanisms, such as appetite suppression, metabolic promotion, reduced fat absorption, increased lipolysis, and decreased lipogenesis (Payab et al., 2020). Among herbal medicines, *Ephedra* is a representative sympathetic stimulant drug. Sympathetic stimulants represent a common type of anti-obesity drugs and can be categorized as appetite suppressants, similar to norepinephrine (Coulter et al., 2018). BP is also a sympathomimetic drug used as an anti-depressant but has an appetite-reducing and weight loss properties and is used alternatively as an anti-obesity drug in combination with naltrexone. (Billes et al., 2014; Barrea et al., 2020). Recently, several studies have investigated the interaction between gut microbiota and the effects of herbal medicines. Gut microbiota digests herbal medicines into active small molecules, which are easily absorbed and have strong physicochemical activities (Wang et al., 2011; Xing et al., 2014; Van Rymenant et al., 2018). Moreover, herbal medicines regulate the composition of gut microbiota and microbe-derived materials, such as short-chain fatty acids (SCFAs), lipopolysaccharides, and hippuric acid, which can induce physiological changes in the host (Chang et al., 2015; Xing et al., 2015; Xu et al., 2015; Chen et al., 2017; Zhang et al., 2018). However, the anti-obesity effect of herbal medicines, such as in sympathetic stimulants, *via* interactions with gut microbiota remains unclear. Therefore, we aimed to identify the influence of ES on gut microbiota by comparing it with bupropion (BP), a different anti-obesity drug.

We hypothesized that ES can modulate the host gut microbiota, which is correlated with body weight and fat accumulation. Therefore, we investigated the anti-obesity effect and gut microbial changes after ES supplementation in a high-fat diet (HFD) model compared with the synthetic drug (BP). This research may provide insights into the application of herbal medicines for the treatment of obesity. To the best of our knowledge, this is the first study that assesses the effects of drugs and herbal medicines on obesity and gut microbiota simultaneously.

## 2 Materials and methods

### 2.1 Botanical drug preparation

*Ephedra sinica* Stapf [Ephedraceae; Ephedrae Herba] (ES) was obtained from Dongguk University Ilsan International Hospital (Goyang, Korea). After washing with distilled water (DW) and oven-dying for 12 h, 500 g of ES was boiled in 4 L of DW for 4 h and filtered using a 300-mesh filter (50 µm). The water extract was concentrated using a vacuum rotary evaporator following lyophilization at 70^°^C and stored at 20°C for future use. Previous studies confirmed the chemical profile of ES with a major component as a standard (Kim et al., 2014; Wang et al., 2017).

### 2.2 Animal studies

Six-week-old male C57BL/6 mice obtained from DBL Inc. (Eumseong-gun, Republic of Korea) were acclimatized for 1 week in a controlled environment with a 12 h light/dark cycle at 25°C, 50%–60% relative humidity, and a chow diet (Purina Irradiated Laboratory Rodent Chow, Purina Korea, Seoul, Republic of Korea). The animal study was approved by the Institutional Animal Care and Use Committee (IACUC-2019–06,187) of Dongguk University Ilsan Hospital and was performed according to the Guide for the Care and Use of Laboratory Animals (National Research Council, 2011).

To assess the efficacy of anti-obesity drug treatment in HFD-induced obese mice, the mice were randomly divided into four groups: control group (NOR), HFD, HFD + BP, and HFD + ES; *n* = 6–8 per group. The mice in the NOR group were fed a 10% fat control diet (D12450B, Research Diets Inc., New Brunswick, NJ, United States), whereas the other groups were fed a 60% HFD (D12492, Research Diets Inc.) for 4 weeks. Mice in the HFD + BP and HFD + ES groups received BP (30 mg/kg/day) and ES (150 mg/kg/day), respectively, *via* oral gavage. Untreated mice in the NOR and HFD groups were orally administered water. Treatments were administered five times a week for eight weeks.

Body weight was measured weekly. Following termination of the experimental period, all animals were fasted overnight. Thereafter, they were sacrificed with a combination of Zoletil (tiletamine-zolazepam, Virbac, Carros, France) and Rompun (xylazine-hydrochloride, Bayer, Leverkusen, Germany) (1:1, v/v). Blood samples were collected from the central aorta and rapidly transferred to a BD Vacutainer (BD, Franklin Lakes, NJ, United States). Visceral, subcutaneous, and perigonadal adipose tissues were quickly excised, washed in ice-cold PBS (pH 7.4), dried, and weighed.

### 2.3 16S rRNA gene amplicon sequencing analysis

Fresh fecal samples were collected 1 week before sacrifice and stored at −80 °C until use. Metagenomic DNA was extracted from samples using a QIAamp stool DNA mini kit (QIAGEN, Hilden, Germany), according to the manufacturer’s instructions. The V1-V2 region of 16S rRNA genes was amplified with polymerase chain reaction using universal primers (8F and 338R) with barcode sequence for multiplexing reads of each sample. Sequencing reactions were performed using an Ion Torrent PGM system (Thermo Scientific, Wilmington, DE, United States), according to the manufacturer’s instructions. Raw sequence reads were quality-filtered, and quality-controlled reads were processed for diversity analysis and taxonomy assignment using the Quantitative Insights into Microbial Ecology 2 (QIIME 2) pipeline V2022.2 (Bolyen et al., 2019). Taxonomy assignment was conducted with VSEARCH, using the Silva database (Quast et al., 2012; Rognes et al., 2016). Beta diversity using unweighted UniFrac distances was calculated using 25,795 reads per sample, representing the minimum number of features observed in each sample. PICRUSt was employed to predict the functional metagenomic profiles of microbial communities with reference to the Kyoto Encyclopedia of Genes and Genomes (KEGG) database (Kanehisa et al., 2012; Langille et al., 2013).

### 2.4 Statistical analysis

Data are expressed as the mean ± SEM unless otherwise indicated. Statistical significance of the animal data was evaluated by one-way analysis of variance (ANOVA) followed by Tukey’s post-hoc test. The statistical significance of the microbiota data was evaluated using the Kruskal–Wallis test followed by Dunn’s test. Statistical significance was set at *p* < 0.05. The strength of the relationships between the parameters was assessed using Spearman’s correlation test with R packages.

## 3 Results

### 3.1 Supplementation of BP and ES prevents body weight gain and fat accumulation

**FIGURE 1:**
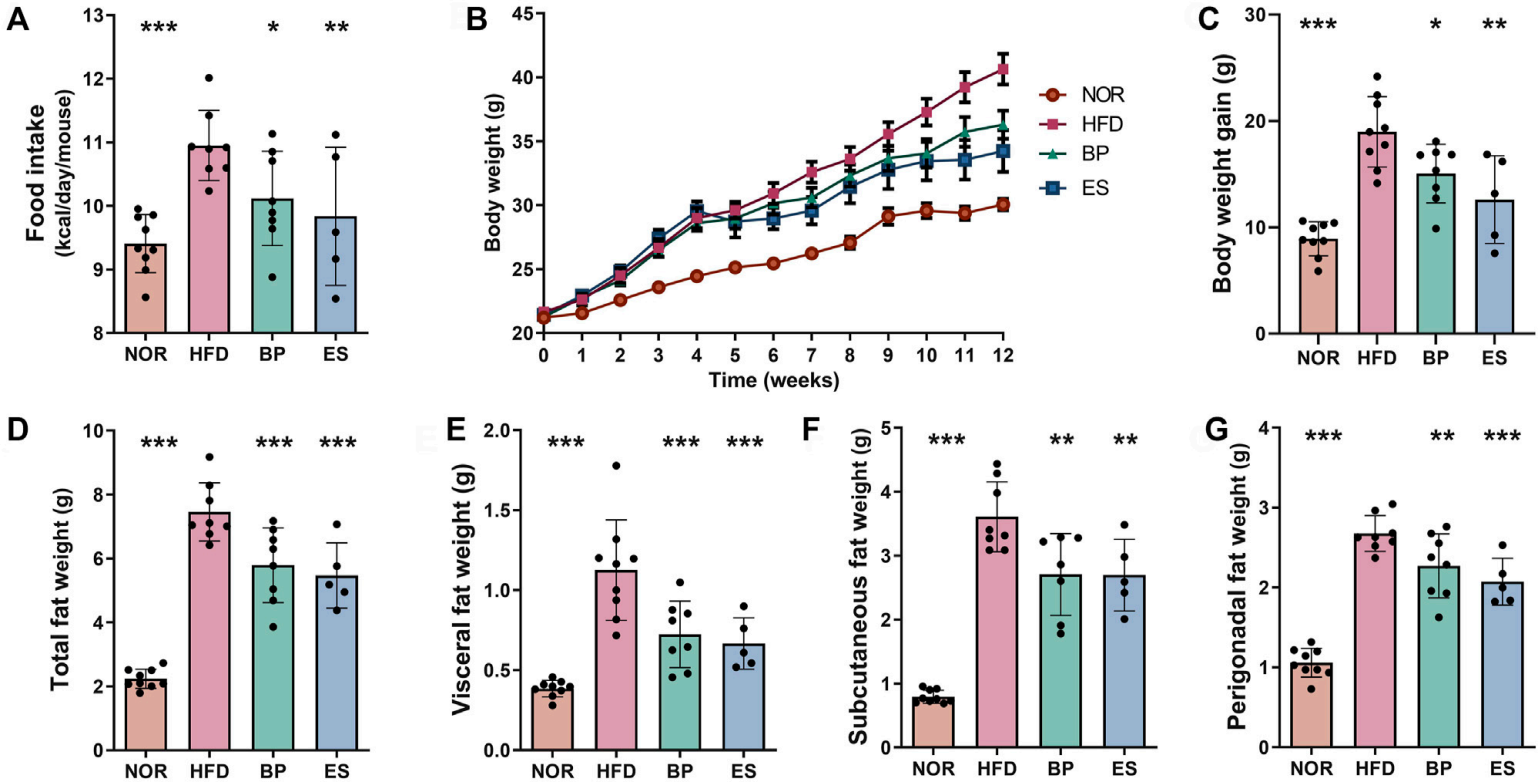
Effect of anti-obesity drugs on weight and fat accumulation in a high-fat diet (HFD)-induced obese model after treatment for 8 weeks. **(A)** Food intake. **(B)** Body weight. **(C)** Body weight gain. **(D)** Total fat weight. **(E)** Visceral fat weight. **(F)** Subcutaneous fat weight. **(G)** Perigonadal fat weight. Data are expressed as the mean ± SEM. Statistical significance was assessed using one-way ANOVA. *p < 0.05, **p < 0.01, ***p < 0.001, vs. HFD. NOR, control group; BP, bupropion; ES, *Ephedra sinica*.

BP and ES are sympathetic stimulant drugs with appetite suppressant and anti-obesity effects (Billes et al., 2014; Kim et al., 2014). Therefore, we established an HFD-induced obese mouse model to investigate the effects of BP and ES on obesity. HFD feeding of mice led to significant increase in food intake, body weight, and fat accumulation compared with the normal diet (NOR) group (Figure 1). BP and ES supplementation considerably decreased food intake by 7.37% and 10.68%, respectively, in HFD-induced obese mice (Figure 1A). Notably, BP and ES supplementation affected body weight and fat accumulation upon HFD feeding. BP and ES supplementation significantly reduced body weight gain by 20.81% and 33.61%, respectively, compared with that in the HFD group (Figures 1B,C). In addition, BP and ES supplementation significantly decreased weights of total fat by 22.31% and 26.65%, respectively, visceral fat by 35.67% and 40.78%, respectively, subcutaneous fat by 25.02% and 25.30%, respectively, and perigonadal fat by 15.17% and 22.56%, respectively, compared with the HFD group (Figures 1D–G). BP and ES supplementation have anti-obesity effects by suppressing appetite and preventing body weight gain and fat accumulation, and although not significant, ES supplementation tended to improve weight-related indicators compared with BP supplementation.

### 3.2 Effect of BP and ES treatment on gut microbiota composition in HFD-induced mice

**FIGURE 2:**
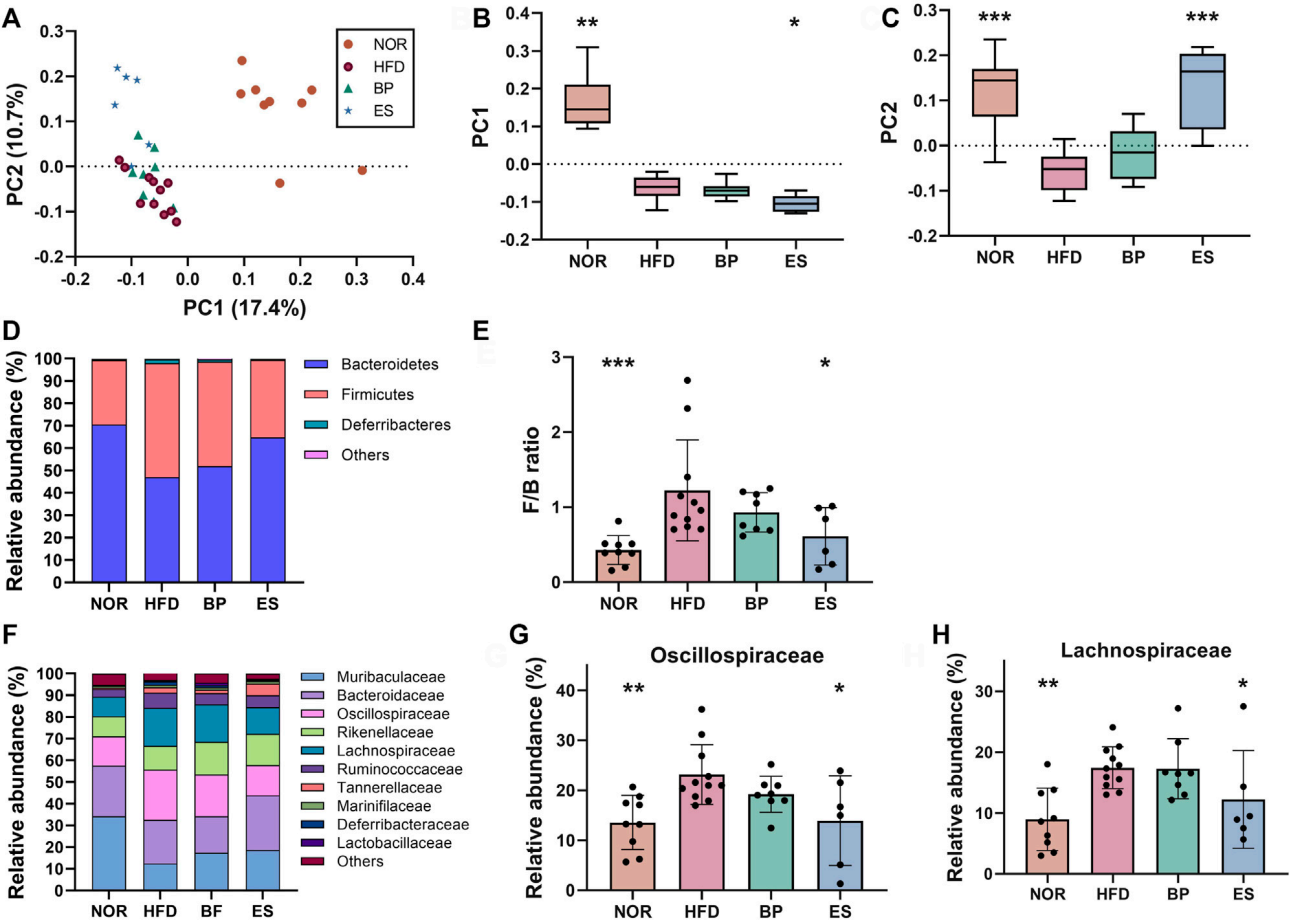
Effect of anti-obesity drugs on the structure of gut microbiota in a high-fat diet (HFD)-induced obese model after treatment for 8 weeks. **(A)** Unweighted UniFrac principal coordinate analysis (PCoA) plot based on the amplicon sequence variants (ASV) abundance of each mouse. **(B)** Box plot of PC1 values in each group. **(C)** Box plot of PC2 values in each group. **(D)** The relative abundance at the phylum level significantly changes with HFD. **(E)** The F/B ratio was calculated as a biomarker of gut dysbiosis. **(F)** The relative abundance at the family level significantly changes with HFD. **(G)** The relative abundance of *Oscillospiraceae* in each group. **(H)** The relative abundance of *Lachnospiraceae* in each group. Values are presented as the mean ± SEM. Statistical significance was assessed using the Kruskal–Wallis test. *p < 0.05, **p < 0.01, ***p < 0.001, vs. HFD. NOR, control group; BP, bupropion; ES, *Ephedra sinica*.

Accumulating evidence suggests that gut microbiota is involved in drug response and metabolism (Vila et al., 2020; Xie et al., 2020). Therefore, gut microbiota might be a potential target for the treatment of obesity. We examined whether BP and ES alter the composition of gut microbiota using 16S rRNA amplicon sequencing of feces. There was no significant difference in alpha diversity such as Shannon index and observed features among groups (Supplementary Figure S1). The principal coordinate analysis (PCoA) plot showed separation among groups, and significance was determined using PC1 and PC2 values on the unweighted UniFrac distance matrix (Figures 2A–C). ES supplementation resulted in a structural shift from the HFD group to the NOR group (Figure 2A). The PC1 value showed no statistically significant difference between the HFD groups (Figure 2B). ES supplementation resulted in a significant difference in PC2 values compared with the HFD group (Figure 2C).

The distribution of bacterial taxa and the relative abundance of bacteria at the phylum level are shown in Figure 2D. The HFD led to a significant increase in the *Firmicutes*/*Bacteroidetes* (F/B) ratio compared with the NOR group (mean ± SD; 0.43 ± 0.19 in NOR vs. 1.22 ± 0.67 in HFD) (Figure 2E). Notably, ES supplementation significantly reduced the F/B ratio compared with that in the HFD group (1.22 ± 0.67 in HFD vs. 0.61 ± 0.38 in ES), however, BP supplementation did not provide a significant result.

The distribution of bacterial taxa and the relative abundance of bacteria at the family level are shown in Figure 2F. HFD feeding led to significant increases in the relative abundance of *Oscillospiraceae* (13.57 ± 5.43 in NOR vs. 23.15 ± 5.95 in HFD) and *Lachnospiraceae* (8.98 ± 5.13 in NOR vs. 17.44 ± 3.42 in HFD) compared with that in the NOR group (Figures 2G,H). Notably, ES supplementation significantly reduced the relative abundance of *Oscillospiraceae* (23.15 ± 5.95 in HFD vs. 13.93 ± 8.98 in ES) and *Lachnospiraceae* (17.44 ± 3.42 in HFD vs. 12.25 ± 8.02 in ES) compared with that in the HFD group; however, BP supplementation did not provide a significant result.

Collectively, ES can restore the gut microbiota dysbiosis such as increased F/B ratio caused by HFD, whereas BP cannot. These results suggest that gut microbiota participates in the response to ES supplementation in terms of recovery from dysbiosis.

### 3.3 Correlation of gut microbiota with body weight and fat accumulation

The metabolic capabilities and disease associations of specific microbial strains are well established (Yan et al., 2020). Different strains within the same species could be differentially affected by the treatment. To detect specific features associated with the anti-obesity effect of drug supplementation, we found amplicon sequence variants (ASVs) that were significantly restored by drug supplementation and analyzed their correlation with body weight and fat accumulation.

**FIGURE 3:**
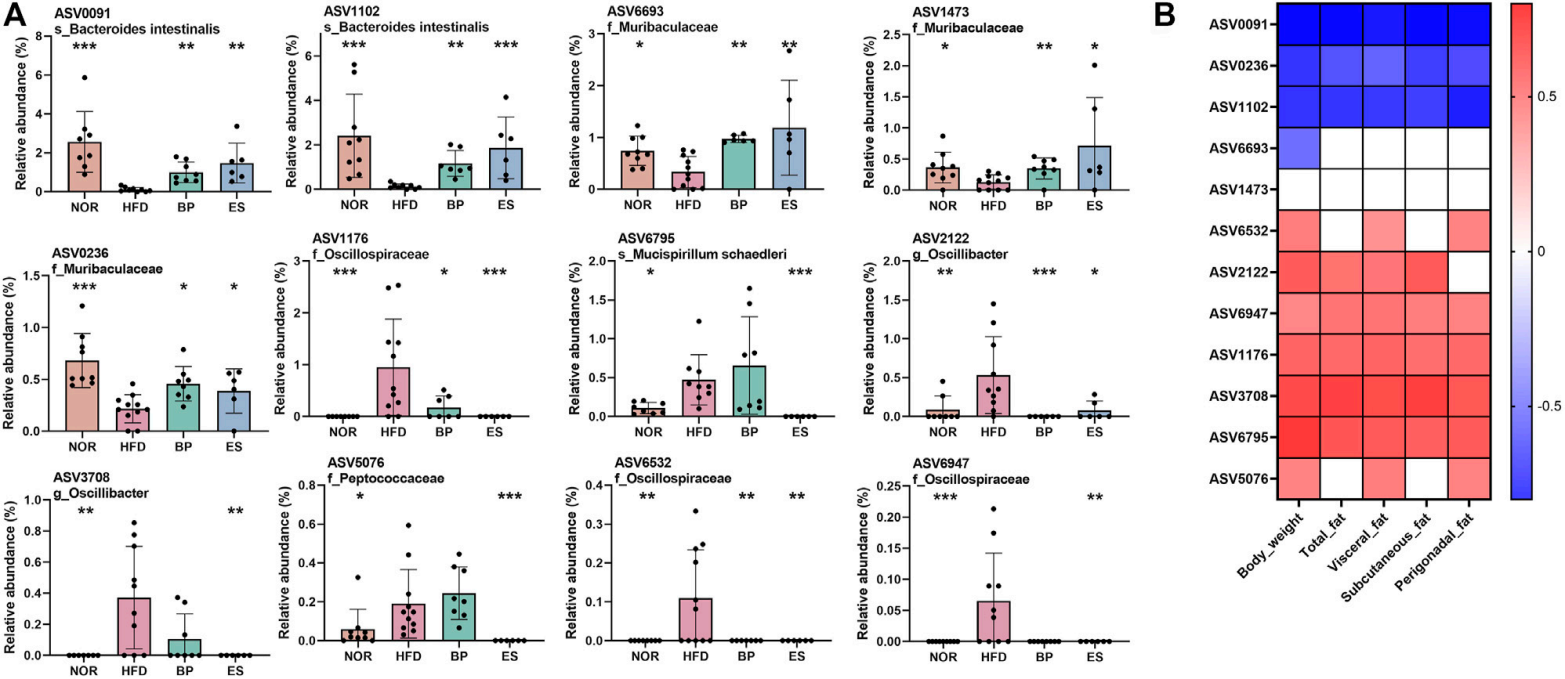
Association of microbiota with anti-obesity drugs and obesity-related parameters. **(A)** Significantly different taxa at the amplicon sequence variant (ASV) level among groups. **(B)** A heatmap generated using Spearman correlation showing the association of significantly different taxa among groups with obesity-related markers. Only correlations with a significance of *p* < 0.05 are represented. Color scale indicates the value of the correlation coefficient. Values are presented as the mean ± SEM. Statistical significance was assessed using the Kruskal–Wallis test. *p < 0.05, **p < 0.01, ***p < 0.001, vs. HFD. NOR, control group; BP, bupropion; ES, *Ephedra sinica*.

Twelve ASVs were found, and their relative abundances are shown in Figure 3A. HFD feeding led to a significant decrease in the relative abundance of five ASVs and an increase in the relative abundance of seven ASVs compared with the NOR group. ES and BP supplementation significantly restored the relative abundance of ASV0091 and ASV1102 assigned to the species *Bacteroides intestinalis*; ASV6693, ASV1473, and ASV0236 assigned to the family *Muribaculaceae*; ASV1176 and ASV6532 assigned to the family *Oscillospiraceae*; and ASV2122 assigned to the genus *Oscillibacter*. Notably, ASV6795 assigned to the species *Mucispirillum schaedleri*, ASV3708 assigned to the genus *Oscillibacter*, ASV6076 assigned to the family *Peptococcaceae*, and ASV6947 assigned to the family *Oscillospiraceae* were significantly decreased by ES supplementation only.

Furthermore, Spearman’s correlation analysis was performed to assess ASVs as biomarkers of body weight and fat accumulation (Figure 3B). We found that 12 ASVs were significantly restored by drug supplementation. Among these, 4 ASVs (ASV0091, ASV0236, ASV1102, and ASV6693) showed a negative correlation and 7 ASVs (ASV6532, ASV2122, ASV6947, ASV1176, ASV3708, ASV6795, and ASV5076) showed a positive correlation with body weight and fat accumulation. In particular, seven ASVs (AS 0091, ASV0236, ASV1102, ASV6947, ASV1176, ASV3708, and ASV6795) showed strong correlation with all fat accumulation indicators.

Collectively, BP, and ES supplementation resulted in specific changes to bacterial ASVs associated with their anti-obesity effects, but the effect of ES supplementation was greater than that of BP supplementation. These results suggest that BP- and ES-induced changes in gut microbiota are linked to the anti-obesity effect.

### 3.4 Functional metagenome prediction analysis

**FIGURE 4:**
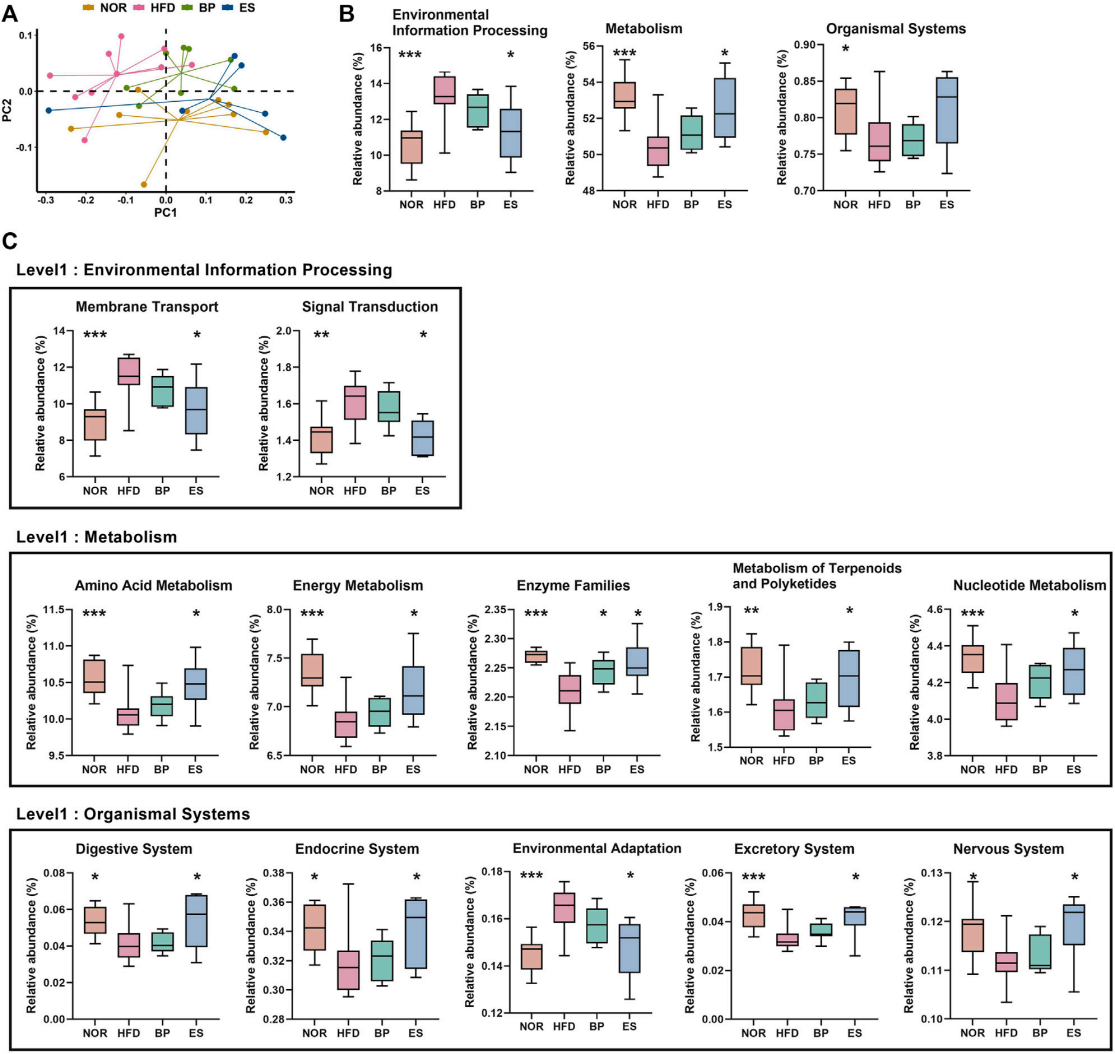
Predictive functional profiling of microbial communities by PICRUSt. **(A)** The principal coordinate analysis (PCoA) generated at the Kyoto Encyclopedia of Genes and Genomes (KEGG) level 3. **(B)** The significantly different pathways at KEGG level 1 among groups. **(C)** The significantly different pathways at KEGG level 2 among groups. Values are presented as the mean ± SEM. Statistical significance was assessed using the Kruskal–Wallis test. *p < 0.05, **p < 0.01, ***p < 0.001, vs. HFD. NOR, control group; BP, bupropion; ES, *Ephedra sinica*.

To evaluate differences in functional attributes of gut microbiota in response to ES supplementation, the predicted functional metagenomic profiles based on the Kyoto Encyclopedia of Genes and Genomes (KEGG) pathways were generated using PICRUSt. The PCoA plot showed separation among groups based on the Bray–Curtis distance matrix of gene content (Figure 4A). Level 1 results showed that HFD led to a significant increase in “Environmental Information Processing” genes and significant decrease in “Metabolism” and “Organismal Systems” genes (Figure 4B). Notably, ES supplementation significantly restored the “Environmental Information Processing” and “Metabolism” genes; however, BP supplementation did not provide a significant result**.**

Level 2 results showed that 12 pathways were associated with the restoration of HFD-induced imbalances by BP and ES supplementation (Figure 4C). We detected that the recovery of genes was related to the pathways of membrane transport, signal transduction, and environmental adaptation by ES treatment, which was increased in the HFD group. In contrast, we detected a recovery in amino acid metabolism, energy metabolism, metabolism of terpenoids and polyketides, nucleotide metabolism, digestive system, endocrine system, excretory system, and nervous system, which decreased after HFD feeding with ES treatment. The decrease in enzyme families after HFD feeding was reversed by both BP and ES.

Level 3 results showed that 32 pathways were associated with the restoration of HFD-induced imbalances by BP and ES supplementation (Supplementary Table S1). The phosphatidylinositol signaling system and tryptophan metabolism were decreased by HFD and restored by BP supplementation. Tetracycline biosynthesis and the insulin signaling pathway were increased by HFD and restored by BP supplementation. Twenty-one pathways, including oxidative phosphorylation and vitamin B6 metabolism, were decreased by HFD and restored by ES supplementation. Nine pathways, including ABC transporters, were increased by HFD and restored by ES supplementation.

Collectively, ES supplementation resulted in restoration of functional profiles including ABC transporters and metabolism-related pathways, in particular, energy metabolism and oxidative phosphorylation. These results suggest that ES induces changes in functional profiles of gut microbiota that may contribute to anti-obesity.

## 4 Discussion

In this study, we hypothesized that some herbal medicines, such as ES, exert anti-obesity effects by inducing changes in gut microbiota. Supplementation with BP and ES prevented body weight gain and fat accumulation. Moreover, ES markedly recovered perturbation of overall composition of gut microbiota, whereas BP and ES considerably recovered members of the gut microbiota, correlated with body weight and fat accumulation, at the ASV level. Only ES significantly recovered predicted pathways, such as environmental information processing and metabolism. These results indicated that the anti-obesity effects of BP and ES are related to the regulation of gut microbiota and that ES has more influence on the modulation of gut microbiota than BP.

In our study, we have discovered that gut microbiota responds to ES supplementation in terms of recovery from dysbiosis but not to BP supplementation. Interest in the association between gut microbiota and obesity treatment is increasing because gut microbiota is involved in drug response and metabolism (Vila et al., 2020; Xie et al., 2020). In addition, recent studies have reported changes in gut microbiota composition following treatment with anti-obesity drugs such as metformin, nuciferine, and orlistat in animal models and clinical trials (Pascale et al., 2019; Ke et al., 2020; Wang et al., 2020). Raineri et al. investigated the effects of four anti-obesity drugs (tacrolimus/FK506, BP, naltrexone, and sibutramine), alone and in combination, on the gut microbiota of obese female rats (Raineri et al., 2022). BP alone had no significant weight change effect and did not affect the changes in gut microbial diversity. The BP and naltrexone combination resulted in increased *Bacteroidetes* and oxidative phosphorylation pathways and decreased the ABC transporter pathway. In our study, although BP exerted anti-obesity effects, such as suppressing appetite and reducing body weight gains and fat accumulation, it did not affect the overall composition of the gut microbiota. Considering our results and those of previous studies, BP alone is insufficient to affect gut microbiota.

A previous study investigated the influence of ES on the composition of gut microbiota in women with obesity (Kim et al., 2014). The impact of ES differed in each subject owing to individual differences in the gut microbiota. However, some bacteria, including *Akkermansia* and *Lactobacillus*, correlated with body weight, body mass index, and body fat percentage after ES consumption. In the current study, we investigated the influence of ES on the composition of gut microbiota in HFD-fed mice. We found that ES changed the overall composition of gut microbiota, and in particular, the F/B ratio-increase due to HFD was also restored. The F/B ratio has been used as a marker of gut microbial dysbiosis and an indicator of obesity (Magne et al., 2020; Stojanov et al., 2020). *Firmicutes* includes numerous known SCFA-producing bacteria, and an increase in the F/B ratio is expected to increase the efficiency of polysaccharide fermentation to SCFA (Louis and Flint, 2009). SCFAs play an important role in various physiological processes, such as host metabolism, gut integrity, glucose homeostasis, lipid metabolism, appetite regulation, and immune function (Morrison and Preston, 2016). However, SCFAs also act as an energy source and increased SCFA production is linked to an increased capacity to harvest energy from the diet (Murphy et al., 2010). Several human studies have shown that the F/B ratio and fecal SCFAs are positively associated (Fernandes et al., 2014; Rahat-Rozenbloom et al., 2014; Goffredo et al., 2016).

In our study, we have discovered that ES induced changes in gut microbiota, and functional profiles of gut microbiota are linked to the anti-obesity effect of ES. *Firmicutes* possess more ABC transporter and phosphotransferase systems compared with *Bacteroidetes* (Mahowald et al., 2009). ABC transporters in *Firmicutes* are often located adjacent to genes encoding glycoside hydrolases, and these two groups may be co-regulated and function together (Rey et al., 2010; Koropatkin et al., 2012). The obese microbiome showed a high enzyme profile of glycoside hydrolases and other enzymes responsible for transport (ABC transporters) and metabolism (α- and β-galactosidases) of products of glycoside hydrolases, resulting in SCFA production (Turnbaugh et al., 2006). We observed a significant increase in the ABC transporter pathway in the HFD group. Consistent with our results, an increased F/B ratio and ABC transporters pathway have been observed in obese and overweight Italian adults (Palmas et al., 2021). Hou et al. observed an association between increased ABC transporter pathway activity and obesity (Hou et al., 2017). Notably, ES supplementation restored the ABC pathway. Our results agreed with previous research that suggested that ES supplementation may have contributed to the modulation of energy harvesting by regulating the ABC transporter pathway and SCFA production, accompanied by restoration of the F/B ratio.

Additionally, we found metabolism-related pathways restored by ES treatment, in particular, energy metabolism and oxidative phosphorylation. Oxidative phosphorylation, also known as the electron transport chain, contributes to ATP synthesis and energy transduction (Nath and Villadsen, 2015). The KEGG pathway for oxidative phosphorylation includes the electron transport chain complexes of NADH dehydrogenase. NADH is a fundamental mediator of energy metabolism (Berger et al., 2004). The pharmacological activation of NADH oxidation has been suggested as a new therapy for the treatment of metabolic syndrome (Hwang et al., 2009). Consistent with our results, exercise enriched the oxidative phosphorylation pathway and reduced fat mass in HFD mice (Lai et al., 2018). *Bupleuri radix* extract also enhanced oxidative phosphorylation and improved lipid disorders in HFD mice (Wu et al., 2021). Although the mechanism of involvement of gut microbiota in the anti-obesity effect of ES remains elusive, our results, in combination with the previous literature, suggest that ES supplementation establishes a gut microbiota community that is adept at oxidative phosphorylation, which may contribute to energy harvesting and reducing fat accumulation. Further confirmation of the role of this pathway in the gut microbiota and the anti-obesity effects of ES remains unexplored.

The ASV with a strong negative correlation between body weight and fat accumulation was assigned to *Bacteroides intestinalis,* which was decreased in the HFD group and restored in both the BP and ES supplementation groups. *Bi73*, a novel gene encoding endoxylanase and esterase, has been identified in transcriptional analysis and is highly upregulated by *B. intestinalis* DSM 17393 (Zhang et al., 2014). Recently, molecular and biochemical analyses have reported a novel trifuctionalendoxylanase/endoglucanase/feruloyl esterase and feruloyl acid (FA)-producing *B. intestinalis* DSM 17393 (Zhang et al., 2022). FA is a plant phenolic acid that is abundant in grains and vegetables (Zhao and Moghadasian, 2008; Molinari et al., 2009). FA suppresses obesity and obesity-related metabolic syndromes in HFD-induced obese mice (Wang et al., 2018). FA also has beneficial effects on diabetes by suppressing oxidative stress, increasing plasma insulin levels, and lowering blood glucose (Roy et al., 2013). Further studies to determine whether *B. intestinalis* is increased by BP and ES supplementation and exerts anti-obesity effects *via* the generation of FA will help better elucidate the mechanism of obesity treatment.

Additionally, an ASV with a strong positive correlation between body weight and fat accumulation was assigned to *Mucispirillum schaedleri*. Consistent with our results, *M. schaedleri* increased in the HFD feeding model and positively correlated with body weight and body fat content (Ussar et al., 2015; Lee et al., 2018; Du et al., 2021). *M. schaedleri* has also been identified as a pathogen associated with intestinal inflammation and oxidative stress (Berry et al., 2015; Loy et al., 2017). Moreover, the development of CD-like diseases is triggered by the presence of *M. schaedleri* (Caruso et al., 2019). We suggest that the increased *M. schaedleri* in HFD serves as a marker of obesity-related dysbiosis, and reduced *M. schaedleri* by ES supplementation is an indicator of the anti-obesity effect of ES. However, further research is needed to elucidate the causality of the relationship between *M. schaedleri* and obesity.

In summary, we investigated the effects of ES on gut microbiota, body weight, and fat accumulation. Our results demonstrated that ES supplementation had an anti-obesity effect, and the potential mechanisms could be due to restoration of gut microbiota dysbiosis related to body weight and fat accumulation. These findings provide evidence for the application of ES as a therapeutic herbal agent for ameliorating obesity, and gut microbiota could be a target for the anti-obesity effect. Nevertheless, preparations derived from Ephedra has safety concerns. *Ephedra* can cause a quickened heartbeat and elevated blood pressure (Soni et al., 2004). The limitations of this study include that we investigate the effect of ES with only single dose study. The clinical application of *Ephedra* as an anti-obesity drug requires careful attention and approach, and sufficient discussion is needed on dose and side effects test. The limitations of this study also include the lack of well-known positive controls; further studies involving positive controls will help confirm the effect of ES on gut microbiota.

## Data Availability

The raw sequencing data presented in this study are deposited in the DNA Data Bank of Japan under accession number DRA013284.

## Abbreviations

ASVs, amplicon sequence variants; BP, Bupropion; ES, Ephedra sinica Stapf; F/B, Firmicutes/Bacteroidete; FFA, free fatty acid; HFD, high-fat diet; KEGG, Kyoto Encyclopedia of Genes and Genomes; LDL, low-density lipoprotein; PCoA, principal coordinate analysis; SCFAs, short-chain fatty acids.
