# Supplementary material for: Impact of the herbal medicine, Ephedra sinica stapf, on gut microbiota and body weight in a diet-induced obesity model
Source: Front Pharmacol. 2022 Nov 15;13:1042833. doi: 10.3389/fphar.2022.1042833 (PMC9706310; doi:10.3389/fphar.2022.1042833)
Supplement: Supplementary file 2 [file Image1.pdf]

Supplementary Material

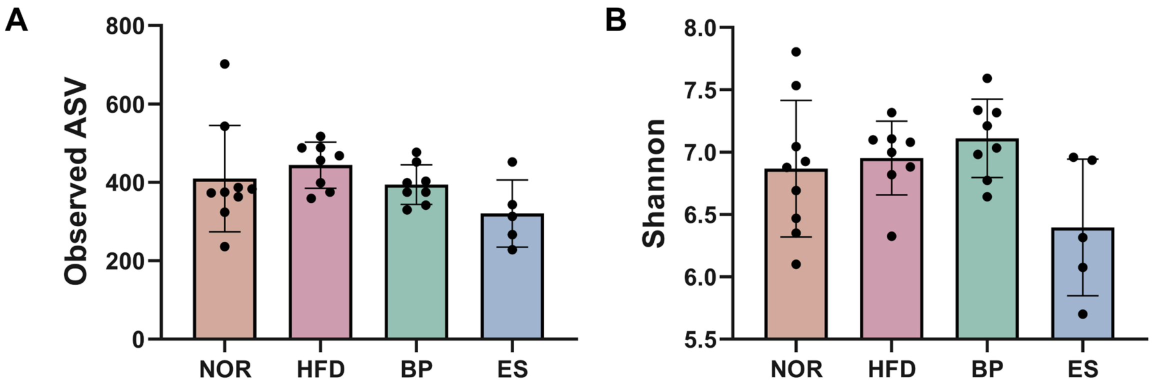

Supplementary Figure 1. Effect of anti-obesity drugs on the alpha diversity of gut microbiota in a high-fat diet (HFD)-induced obese model after treatment for 8 weeks. (A) Count of observed amplicon sequence variant (ASV) in each group. (B) Shannon index in each group. Statistical significance was assessed using the Kruskal–Wallis test. *P <0.05, **P <0.01, ***P <0.001, vs HFD. NOR, control group; BP, bupropion; ES, Ephedra sinica.
